# Supplementary material for: Conservation laws and the foundations of quantum mechanics
Source: Proc Natl Acad Sci U S A. 2023 Oct 2;120(41):e2220810120. doi: 10.1073/pnas.2220810120 (PMC10576110; doi:10.1073/pnas.2220810120)
Supplement: Supplementary file 1 — Appendix 01 (PDF) [file pnas.2220810120.sapp.pdf]

Supporting Information for

Conservation laws and the foundations of quantum mechanics

Yakir Aharonov, Sandu Popescu and Daniel Rohrlich

Corresponding Author Popescu.
E-mail: s.popescu@bristol.ac.uk

This PDF file includes:

Supporting text
SI References

Supporting Information Text

Supplementary Information I

The proof of the superoscillatory nature of $f(x)$ over the range of $|x| \leq O(N^{1/2-\epsilon})$ where ϵ is an infinitesimally small, positive constant has been given in (1). We include it here for convenience.

Theorem: In the limit of large N, for any $|x| \leq \mathcal{O}(N^{1/2-\epsilon})$ with ϵ positive and infinitesimally small, and $\alpha > 1$, the function $f(x)$ can be approximated by

$$f(x) \approx e^{i\alpha x}. \quad [1]$$

Proof. Let us express f by using its absolute value and phase:

$$\begin{aligned} f(x) &= \left(\frac{1+\alpha}{2}e^{i\frac{x}{N}} + \frac{1-\alpha}{2}e^{-i\frac{x}{N}}\right)^N = \\ &\left(\cos\frac{x}{N} + i\alpha \sin\frac{x}{N}\right)^N = \\ &\left(1 + (\alpha^2 - 1)\sin^2\frac{x}{N}\right)^{\frac{N}{2}} e^{iN\arctan(\alpha\tan\frac{x}{N})} \end{aligned} \quad [2]$$

First, let us consider the absolute value of f. We have

$$\begin{aligned} 1 \quad &\leq \quad |f(x)| = \left(1 + (\alpha^2-1)\sin^2\frac{x}{N}\right)^{\frac{N}{2}} \\ &\leq \quad \left(1 + (\alpha^2-1)\frac{x^2}{N^2}\right)^{\frac{N}{2}} \\ &\leq \quad \left(1 + (\alpha^2-1)\frac{1}{N^{1+2\epsilon}}\right)^{\frac{N}{2}} \to 1 \end{aligned} \quad [3]$$

where in the last inequality we have used $|x| \leq \mathcal{O}(N^{1/2-\epsilon})$ and where the final limit is standard.

Let us consider now the phase. For the approximation below all we require is $\frac{x}{N} << 1$, which can be fulfilled for $|x| = \mu N$ where $\mu << 1$ is an arbitrary fixed constant. Then, using first order approximation

$$N\arctan(\alpha\tan\frac{x}{N}) \approx N\arctan(\alpha\frac{x}{N}) \approx N\alpha\frac{x}{N} = \alpha x. \quad [4]$$

$\square$ *QED*

Note that the region where $f(x)$ looks like a plane wave of wavenumber α is of order $O(N^{1/2-\epsilon})$ and that the limitation to this range follows from the behaviour of the absolute value of f. Indeed, for $|x|$ of order $O(N^{1/2})$ the absolute value of f starts increasing; in particular, for $|x| = N^{1/2}$ we get $|f(x)| \to e^{\frac{\alpha^2-1}{2}}$ as we can readily see by using this value in (3). On the other hand, the phase continues to superoscillate on a much larger region, of order N.

Supplementary Information II

For simplicity, the preparation evolution described in eq (20) of the main text, (given below again for convenience)

$$|0\rangle_p|\Phi\rangle_P \to |\chi\rangle_{p,P} = \sum_{m=-N}^{N} c_m|m\rangle_p|\Phi - m\rangle_P \quad [5]$$

has been described only as it acts on $|0\rangle_p|\Phi\rangle_P$, our particular initial state of interest. Here we would like to show that this evolution can be implemented by a unitary that conserves angular momentum. For this we have to define the action of evolution operator on all the states in the Hilbert space of the particle and preparer.

Suppose that we have a transformation $\hat{U}$ such that

$$\hat{U}|0\rangle_p|k\rangle_P = \sum_{m=-N}^{N} c_m|m\rangle_p|k-m\rangle_P \quad [6]$$

with $-\infty < k < \infty$.

Then, for any initial wavefunction $|\tilde{\Phi}\rangle_P$ of the preparer we have

$$\hat{U}|0\rangle_p|\tilde{\Phi}\rangle_P = \sum_{m=-N}^{N} c_m|m\rangle_p|\tilde{\Phi} - m\rangle_P \quad [7]$$

as we can easily see if we insert in (7) the angular momentum decomposition of $|\tilde{\Phi}\rangle_P$ and use (6). In other words, having a transformation $\hat{U}$ that fulfils (6) is sufficient for implementing the transformation (5) that we desire.

It is convenient to write the transformation (6) in total and relative angular momentum variables, $\hat{L}_t = \hat{L}_p + \hat{L}_P$ and $\hat{L}_r = \hat{L}_p - \hat{L}_P$. In these new variables the transformation reads

$$\hat{U}|-k\rangle_r|k\rangle_t = \sum_{m=-N}^{N} c_m|m-k\rangle_r|k\rangle_t = |\xi_{-k,k}\rangle_r|k\rangle_t \quad [8]$$

where $|\xi_{-k,k}\rangle_r = \sum_{m=-N}^{N} c_m|m-k\rangle_r$.

We can now extend the transformation (8) to a full, angular momentum conserving, unitary. For this we have to extend it to all possible initial states $|n\rangle_r|k\rangle_t$ with $-\infty < n, k < \infty$, i.e. to define its action on an entire basis of states. All we have to do is to define

$$\hat{U}|n\rangle_r|k\rangle_t = |\xi_{n,k}\rangle_r|k\rangle_t \quad [9]$$

with the states $|\xi_{n,k}\rangle_r$ are arbitrary, except $|\xi_{n-k,k}\rangle_r$ which is fixed by (8), and obey the orthogonality conditions

$${}_r\langle\xi_{n,k}|\xi_{n',k}\rangle_r = \delta_{n,n'}. \quad [10]$$

We can now see that for all the initial states $|0\rangle_p|k\rangle_P$ the transformation $\hat{U}$ is angular momentum conserving and takes orthogonal states into orthogonal states, as required by a unitary. Since for any fix total angular momentum k a single state of relative momentum, namely $|\xi_{-k,k}\rangle_r$ is fixed by our desired transformation (5), we have plenty (infinite) of liberty to chose the states $|\xi_{n,k}\rangle_r$ with $n \neq -k$ so that together with $|\xi_{-k,k}\rangle_r$ they form a basis. Clearly then, the transformation (9) is unitary (since it transforms the orthonormal basis $\{|n\rangle_r|k\rangle_t\}$ into the orthonormal basis $\{\xi_{n,k}\rangle_r|k\rangle_t\}$,) it is conserving the total angular momentum and it implements on the state $|0\rangle_p|\Phi\rangle_P$ the transformation we desire.

References

1. Y. Aharonov, S. Popescu and D. Rohrlich, *On conservation laws in quantum mechanics*, PNAS, 118, e1921529118 (2021).
